# Application of Core Processes for Understanding Multiple Concurrent Sexual Partnerships Among Adolescents in Uganda

**DOI:** 10.3389/fpubh.2018.00371

**Published:** 2018-12-21

**Authors:** Judith Nalukwago, Jane Alaii, Bart Van Den Borne, Paul Mukisa Bukuluki, Rik Crutzen

**Affiliations:** ^1^Care and Public Health Research Institute (CAPHRI), Maastricht University, Maastricht, Netherlands; ^2^Family Health International 360 (Uganda), Kampala, Uganda; ^3^Department of Social Work and Social Administration, Makerere University, Kampala, Uganda; ^4^Context Factor Solutions, Nairobi, Kenya

**Keywords:** adolescents, multiple concurrent sexual partnerships, determinants, core processes, Intervention Mapping

## Abstract

**Introduction:** Adolescents in Uganda, as in other sub-Saharan countries, engage in sex with multiple concurrent partners, thus placing them at risk for HIV and unplanned pregnancies, but it is not clear why. This study explored why adolescents in Uganda engage in multiple concurrent sexual partnerships (MCSP).

**Methods:** This study used a Core Processes methodology. We used the processes of brainstorming, and identification of evidence and theoretical support, in various phases/steps of intervention planning, to provide possible explanations for adolescent MCSP.

**Results:** Adolescents were found to have limited knowledge of the risks associated with MCSP and perceived a low risk for HIV. Peer influence to engage in MCSP exacerbated the problem among adolescents. Poor communication with sexual partners and parents and societal indifference to multiple sexual partnerships increased permissive attitudes toward infidelity. The unclear adolescent sexual and reproductive health policies hampered access to services, and transactional sexual relationships with older (polygamous) sexual partners increased the HIV risk. Adolescents were found to be more concerned about unplanned pregnancies than HIV risk.

**Discussion:** From the empirical evidence, adolescent health programs in Uganda should incorporate comprehensive sexual health education on HIV and teenage pregnancy risk-reduction strategies. Programs should strengthen parental and community support through enhanced collaborative training on communication with and for adolescents. Forming strategic partnerships with various stakeholders for concerted efforts to address the MCSP problem among adolescents is critical.

## Introduction

Multiple sexual partnering is on the rise among adolescents aged 15–19 in Uganda. National surveys show that in 2016, 2.2% of adolescent females and 6.6% of males reported having two or more sexual partners in the past 12 months compared to 2011 (1.5% for females and 5.4% for males) (1, 2). Moreover, adolescents often have sexual partnerships that overlap for months or years (3–5). These sexual partnerships are labeled as multiple concurrent sexual partnerships (MCSP). MCSP are associated with a heightened risk of sexually transmitted diseases (STDs), particularly HIV/AIDS (6–9). It is not yet clear why adolescents in Uganda engage in MCSP, which can lead to health problems of unplanned pregnancies and ultimately a decreased quality of life. Most adolescent sexual behavior studies in Uganda have focused on HIV prevention in light of condom use, and include limited explanations for MCSP. This study used Core Processes including brainstorming and the identification of empirical evidence and theoretical support to explore persistent MCSP among adolescents in Uganda.

MCSP is defined in this study as an overlap of sexual partners in a given time period, with two or more simultaneous sexual partnerships. Adolescents who engage in MCSP increase the risk of acquiring and subsequently exposing partners in their sexual network to HIV (6, 10–13). Many individuals infected with HIV in sub-Saharan Africa have been found to be men and women in stable partnerships, although some have become infected during MCSP within their stable relationship or their partner's engagement in MCSP (14). Thus, in order to design targeted adolescent health programs in Uganda, it is imperative to determine why there are persistent MCSP among adolescents. Identifying determinants for this behavior requires investigating individual, community and environmental factors (15).

This study used a Core Processes methodology to explore why adolescents in Uganda engage in MCSP. Core Processes are a systematic way to answer questions raised in various phases/steps of planning frameworks for program development (e.g., “Why do people in the priority population carry out the (risk) behavior?”) (15). Identification and formulations of these core processes originated from Veen (16) and Lave and March (17) and were further developed by Buunk and Veen (18), Kok et al. (19), Buunk and Van Vugt (20), Buunk and Van Vugt (21), Bartholomew et al. (15, 22–24). Although Core Processes are described in Intervention Mapping (15), they can be applied to any planning framework. So, Core Processes do not form a planning framework on their own, but rather operate as a systematic approach to address questions relevant to problem definition and solutions.

## Methods

### The Core Processes Methodology

This study used the Core Processes methodology described in Intervention Mapping (15) to explore MCSP among adolescents in Uganda. The methodology provides a systematic way to answer questions raised in distinct phases/steps of planning frameworks. The steps described in the methodology are crucial to answer questions in such a way that the chances of adequately addressing the problem with new research at hand are optimized (25). Using Core Processes minimizes the likelihood of achieving an incomplete understanding and thus selecting ineffective solutions because the processes use available evidence before engaging in new research. The Core Processes involve six steps: (1) posing the problem question, (2) brainstorming for possible answers, (3) reviewing empirical literature related to the problem, (4) identifying theoretical support based on the topic, concepts, and general theoretical approaches, (5) identifying and addressing new needs for research on the problem, and (6) proposing answers to the problem. The order of steps in the Core Processes is crucial; brainstorming (step 2) utilizes theoretical and empirical knowledge available within the planning group that can later be combined with empirical findings (step 3) and theoretical support (step 4). As a result of completing the steps, the planners assemble a set of potential answers to the problem from both the theoretical and the empirical literature that fit with, suggest changes for, or add to provisional explanations. The six steps of the Core Processes used in the current study are detailed below.

### Posed the Question of Adolescents Engaging in MCSP

In the introduction, we noted why engaging in MCSP is a health risk. The point of departure of the study was the question “*Why do adolescents in Uganda engage in MCSP?*”

### Brainstorming on Possible Explanations for Adolescent MCSP

In 2017, brainstorming sessions to identify possible determinants and factors explaining why adolescents engage in MCSP were held with various stakeholders and experts in Uganda. Studies argue that brainstorming is an intervention in which individuals and groups adhere to a set of rules while working in sessions designated to generate ideas (26). Brainstorming is conducted to (1) generate as many ideas as possible, (2) avoid criticism of proposed ideas, (3) attempt to combine and improve previously articulated ideas, and (4) encourage generation of “wild” ideas, and (5) record all ideas for future consideration (26, 27). A discussion guide is used to clarify the question flow and the emphasis placed on each question (26, 27). Brainstorming allows people with multiple areas of expertise to come together as a whole greater than the sum of the individual parts (28).

The brainstorming sessions for this study were conducted during quarterly monitoring activities undertaken by Communication for Healthy Communities (CHC), a social and behavior change communication program funded by the United States Agency for International Development and implemented by Family Health International 360 (FHI 360) (29). To focus the brainstorming sessions on topics related to adolescent sexual behaviors and sexual and reproductive health (SRH) needs, a discussion guide was developed based on the study objectives. The questions in the discussion guide focused on underscoring adolescent sexual behaviors (with specific reference to MCSP) and the risks associated with the behaviors, existence and appropriateness of SRH services for adolescents and information sources in the community, the environmental (household, community, structural, or health system) determinants perceived to affect adolescents' ability to take up health services and healthy behaviors, and recommended strategies for addressing adolescent SRH needs and promoting healthy behaviors. The same questions were asked in each session for consistency in generating a pool of explanations for why adolescents engage in MCSP.

**Figure 1:**
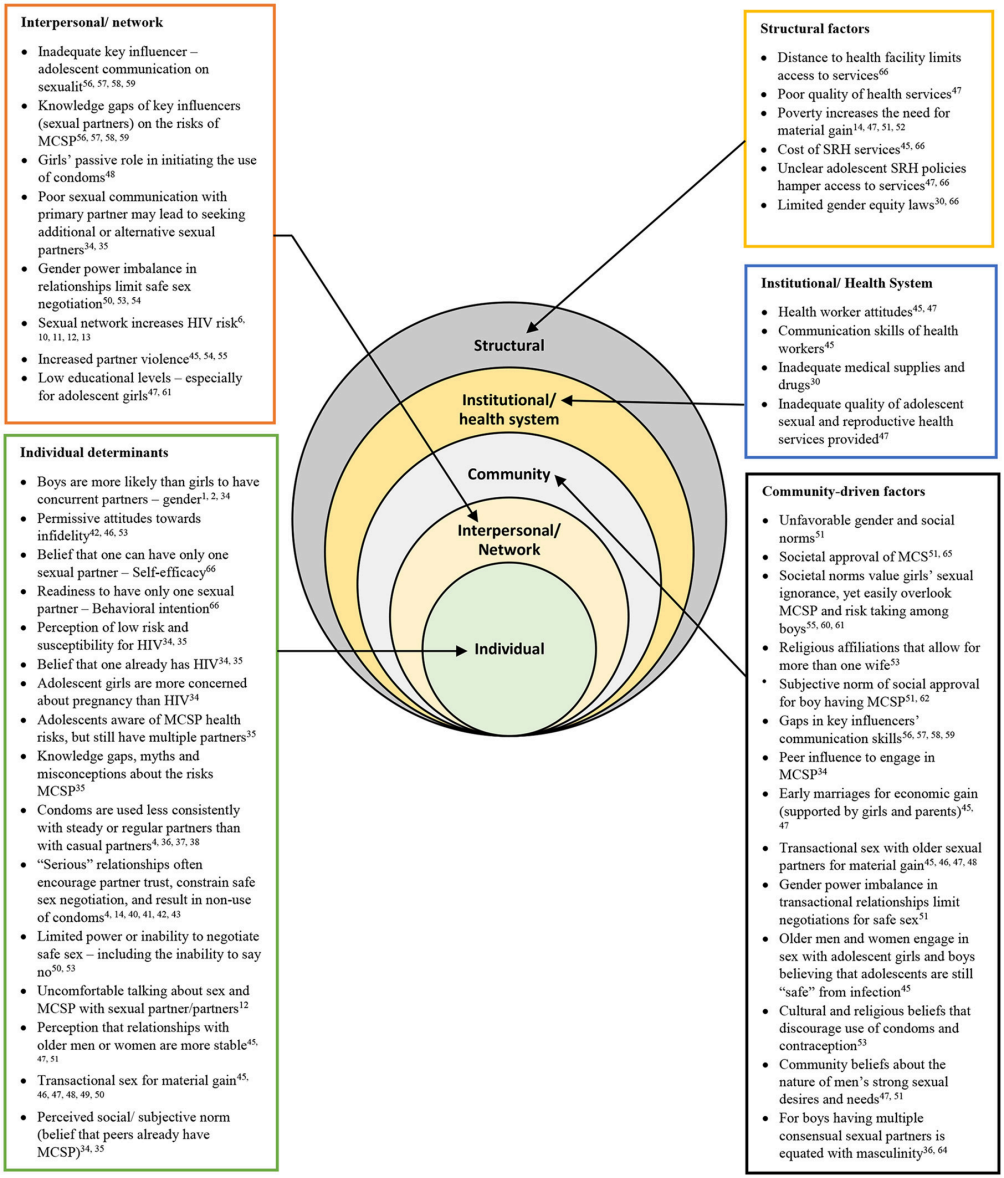
Determinants of multiple sexual partnerships among adolescents at all levels of the Social Ecological Model based on literature findings. Depiction of Social-Ecological Model adapted from Mckee et al. (32) and Kaufman et al. (30). MCSP, Multiple Concurrent Sexual Partnerships; SRH, sexual and reproductive health.

First, separate brainstorming sessions were held with adolescents, community leaders (religious leaders, community health workers, and local council leaders), and experts from the Ministry of Health in Uganda. Based on the outcome of those sessions, brainstorming sessions were held with additional experts including behavioral scientists, health promotion practitioners, sociologists, and monitoring-and-evaluation experts to draw on their practical experience in addressing adolescent health issues in various contexts in Uganda. The additional experts who participated in the brainstorming sessions were selected based on their knowledge of Uganda, expertise working with adolescents, and experience in designing and implementing adolescent health promotion programs. In total, five brainstorming sessions were conducted: two with adolescents, two with community leaders, and one with additional experts. Each session had between 8 and 12 participants, and lasted an average of 1 h. Studies indicate that attempts to understand the process of behavior change or to develop an intervention should consider all levels of influence and related variables from individual to structural (30, 31). Therefore, based on the Social Ecological Model, the brainstorming sessions put particular emphasis on identifying and classifying behavioral determinants and factors reflected at multiple levels of influence (30, 32, 33) including individual, interpersonal, community, institutional, and structural factors (Figure 1). The adolescents and community leaders were mobilized through community health workers who work with CHC. Sixteen adolescents (eight girls and eight boys), 15 community leaders (eight female and seven male), and eight experts (three female and five male) participated in the brainstorming sessions. To ensure full participation, the sessions for male and female participants were conducted separately. Preliminary answers/explanations on the problem identified during the brainstorming session were written down to guide an extensive literature review on why adolescents engage in MCSP.

### Participants' Informed Consent

Verbal informed consent was sought from each study participant prior to brainstorming sessions. Ethical approval and a waiver of written consent was obtained from the United States of America's federally registered institutional review board of FHI 360, the Protection of Human Subjects Committee, under reference 939934, and in Uganda by the government-accredited Makerere School of Public Health Research Ethics Committee under reference 446. Verbal consent was preferred to minimize the risk of a loss of anonymity because signed informed consents would have been a link to the participants' identity in this study. The consenting process was conducted in both English and a local language that participants understood. The purpose of the study was explained, and participants were given time to ask questions before voluntarily agreeing to participate in the study. Verbal informed consent was documented by recording the date and time when the consent was sought and the signature of the study staff member who obtained consent.

### Reviewed Empirical Literature on Adolescent MCSP

Insights from the brainstorming sessions informed an extensive literature search and review on adolescent MCSP. The literature review included a search of multiple electronic databases including Google Scholar, Cochrane central (via Cochrane library), Wolters Kluwer (via Ovid), PubMed, Emerald, and PsychINFO (via EBSCOhost) to retrieve empirical studies on MCSP. The specific search terms used were multiple sexual partnerships, concurrent partnerships, multiple concurrent partnerships, factors, determinants, correlates, gender, and predictors associated with MCSP. The search specifically focused on, but was not limited to, studies of adolescents. Only studies that highlighted aspects of multiple sexual partnerships and MCSP among adolescents and those that compared adolescent MCSP with adult MCSP were considered. The extensive review of existing literature on MCSP among adolescents focused on learning what is known worldwide including sub-Saharan Africa, East Africa, and Uganda. The researchers identified issues of missing links or what was not known or not clear in the literature about adolescent MCSP. The literature search ranged from 1990 to 2018, and ~120 studies were identified. However, we used 98 studies found to be related to the current study. Studies were selected based on the inclusion criteria of highlighting adolescent MCSP and factors associated with MCSP. We excluded those that explored sexual behaviors other than MCSP among adolescents. Findings from this review are presented in Figure 1 and **Table 3**.

### Identified Theoretical Support

Theoretical support for the study was identified using three main approaches: topic, concept, and general theories (15). The topic approach involved a review of theoretical constructs used in the design of other empirical studies on MCSP included in the literature review. Most of the included empirical studies used theories to provide potential explanations for MCSP. The identified theories were used as the first step to select theoretical support for the present study.

The concepts approach involved scrutiny of ideas generated during the brainstorming sessions and grouping them by aspects of similarity. For precision, related ideas from the brainstorming sessions and empirical literature were renamed using theoretical labels.

The general theories approach involved exploring a vast array of theories that might offer detailed explanations and answers to the problem of adolescent MCSP. The study researchers further explored alternative theoretical frameworks including behavioral, community and environmental theories that had not been assessed through the topic and concept approaches.

### Identified and Addressed New Needs for Research on Adolescent MCSP

From the selected empirical literature, the missing links, or what is not yet known and remains unclear about adolescent MCSP were identified. Specific research questions for further exploration in Uganda were formulated based on the literature review results.

### Proposed Answers to Reducing the Problem of Adolescent MCSP

Using findings from the brainstorming sessions, literature review, and exploration of theoretical support, the researchers listed possible explanations as to why adolescents in Uganda engage in MCSP. These explanations will inform future adolescent health programming to decrease the MCSP behavior. The frequently listed possible explanations of the problem were ranked by order of importance and changeability. Importance refers to the determinants that significantly contribute to the behavior, and changeability refers to determinants that can be changed with available methods (15). The ranking procedure was adapted from Bartholomew-Eldredge et al. (15) as 0 = less important, + = important, and ++ = very important and 0 = hard to change, + = may be changed, ++ = it is changeable. The explanations/answers that appeared in all three theoretical support approaches (topic, concept, and general theories) and suggested practicable interventions to address the problem were ranked with high importance and changeability. Ranking of the possible explanations/answers helped to prioritize determinants and factors for adolescent MCSP to guide targeted adolescent health programs.

It was important that the design-planning group for the program completed the previously described steps of the core processes instead of jumping straight into research. Conducting new research requires resources including time, expertise and money. All available evidence and insight should be used before conducting new research to guide program design. The order of steps following the problem identification (step 1) is crucial: brainstorming (step 2) ensures utilizing the theoretical and empirical knowledge that is available within the planning group, which can later be combined with empirical findings (step 3) and identification of theoretical support (step 4). Taking these steps should improve new research strategies.

## Results

### Findings From Brainstorming Sessions With Stakeholders in Uganda (Step 2 of Core Processes)

Group brainstorming sessions with adolescents indicated that adolescents in Uganda engage in MCSP because of peer pressure, and a lack of basic needs drives them to engage in multiple transactional sexual relationships for money. Although some adolescents suggested that engaging in MCSP was a way of exploring life, others noted that it was morally not okay to have multiple concurrent sexual partners. Some adolescents alleged that occasionally parents encourage them to engage in transactional sex and early marriages to older sexual partners who may be polygamous, which increases their risk for HIV. However, parent-adolescent communication gaps make adolescents fearful of talking openly about sex (Table 1).

**Table 1:** A summary of findings from brainstorming sessions on MCSP with stakeholders.

**Stakeholder category**	**Main issues raised from the brainstorming sessions**
Adolescents	• Peer pressure • Transactional sex • Exploring life • Morally not okay to have multiple concurrent sexual partners • Poor parent-adolescent communication
Community leaders and policymakers	• Parents' laxity in disciplining children • Poor family background creates an inappropriate environment for adolescent sexual behavior • Some parents can be bad examples to adolescent children • Domestic violence • Churches provide opportunities for risky adolescent sexual behavior • Poor parent-adolescent communication • Inadequate policies limiting adolescent sexual activities
Other experts in additional fields (behavioral scientists, health promotion practitioners, sociologists)	• The belief that is okay to have many partners • Adolescents believe it is good to experiment with MCSP • MCSP is seen as a sign of masculinity, braveness and being a man • Adolescents and parents do not see the risk of MCSP • Poverty • Limited and inconsistent use of condoms in MCSP

*MCSP, Multiple Concurrent Sexual Partnerships*.

Community leaders and policymakers from the Ministry of Health indicated parents' laxity and lack of discipline for their children, and fading community support for disciplining children all contribute to the problem of adolescent MCSP. They further alluded to poor family backgrounds that create an inappropriate environment for adolescent sexual behavior. For instance, some parents were cited to be setting a bad example by dressing in a manner considered to be indecent, while domestic violence reportedly drove adolescents away from homes. Parent-adolescent communication was mentioned as lacking in homes, and inadequate government policies for prohibiting early marriages to limit adolescent sexual activities. Away from home, some churches were noted to expose adolescents to sexual risks during unchaperoned activities such as night prayers and concerts.

Brainstorming sessions were also held with additional experts, including behavioral scientists, health promotion practitioners, sociologists, and monitoring, and evaluation specialists. Findings from these sessions indicated that individuals' attitudes and beliefs about masculinity, such as “it is okay for a man to have many sexual partners,” encourage male adolescents to engage in MCSP. Male adolescents often believe that it is good to experiment with many sexual partners because they see it as a sign of braveness, and “being a man.” The experts further noted that adolescents lacked the confidence to commit to only one sexual partner. In this sense, self-confidence is often affected by other factors including an individual's environment (e.g., difficult family situation) and need for means of survival (e.g., extreme poverty). They noted that adolescents and their parents do not often perceive or they may not be aware of the risks of engaging in MCSP. Poverty was also indicated as a factor that makes adolescents engage in transactional sex, including MCSP. Limited and inconsistent use of condoms among adolescents was highlighted as a challenge in multiple sexual relationships. Table 1 summarizes the findings from the brainstorming sessions with stakeholders.

### Findings From the Review of Empirical Literature (Step 3 of Core Processes)

To synthesize empirical literature findings on adolescent MCSP, the main determinants, and levels of influence were categorized using the Social Ecological Model (30, 32, 33) as indicated in Figure 1. The findings indicate that adolescents often report having two or more sexual partners, and boys are more likely to have concurrent sexual partners than girls (1, 2, 38). Individual determinants drawn from the literature indicate that adolescents perceive they have a low risk and susceptibility to HIV (34, 38). Others engage in MCSP with the belief that they already carry HIV/AIDS (34, 38). Studies found that condoms are used less consistently in relationships with MCSP, which increases the risk of HIV spreading to the adolescent's sexual network (4, 42–45). In addition, condoms were used less consistently with steady or regular sexual partners than with casual sexual partners (4, 44, 45). Adolescent girls who perceive to be in “serious” relationships are socially pressured to trust their partners, which constrains their ability to negotiate safe sex and results in the non-use of condoms (4, 14, 43, 46–49). Although adolescents were found to be aware of the risks associated with MCSP, they continue to engage in these relationships (34). Adolescent girls were found to be more concerned about unplanned pregnancy than HIV risk (38). Adolescents were also found to engage in transactional sexual relationships with older men and women for material gain or money, and the belief that relationships with these partners are stable (40, 50–55). This is an indication that adolescents may have inadequate knowledge of the consequences of MCSP.

Poor communication with the primary sexual partner intersects between individual and interpersonal determinants. Studies indicated that poor sexual communication with the primary sexual partner might lead to seeking additional or other sexual partners (34, 38). Adolescents who engage in MCSP with older sexual partners often experience gender power imbalances that limit their power to negotiate for safe sex practices and may feel uncomfortable talking to their sexual partner about sex, and MCSP in general (55–58). Girls have been found to take a more passive role in initiating the use of condoms since they believe that boys/men need more sex than girls/women (52). Furthermore, studies report that concurrent relationships with gender power imbalances can be characterized by physical intimate partner violence (40, 58, 59). However, knowledge gaps on issues of sexuality and MCSP and poor communication skills of key influencers such as sexual partners, parents, and peers were found to limit open discussion of sexuality, thereby precipitating MCSP among adolescents. Parents were found to have difficulty in finding the right place and time to communicate with their children about sexuality. This is because parents often feel inadequately informed, embarrassed, and uncomfortable discussing topics of sexuality (60–63).

Adolescents were found to engage in MCSP because they believed that peers already had multiple sexual partners (34, 38). This is an indication that the social environment of adolescents ought to be critically scrutinized to underscore community-driven factors that encourage MCSP. Studies indicate significant societal approval of MCSP, especially for boys/men. Societal norms value sexual ignorance for girls while overlooking MCSP and sexual risk-taking for boys, both of which contribute to the problem (58, 62, 64, 65). A man's infidelity is commonly accepted and is often viewed as something inevitable that a woman must simply expect and learn to tolerate (55, 66). With this societal approval, adolescent boys were found to have more permissive attitudes toward infidelity than girls (50, 55, 57, 67). Older men and women were found to advance transactional sexual relationships with adolescent girls and boys with the belief that adolescents are still “safe” from infection (40). Similarly, early marriages to older men for material gain (precipitated by some parents and adolescent girls), increase the girls' risk for HIV and teenage pregnancy because of their inadequate reproductive health education and subordinate position when negotiating safe sex with adult sexual partners (40, 51). However, studies have also found that previously married adolescent girls are more likely to have multiple sexual partnerships compared to married or never married counterparts (3). Community beliefs about the nature of men's strong sexual desires and needs create an oblique view of having concurrent sexual partners for boys, which is often seen as a sign of masculinity (42, 51, 55, 68, 69). Cultural and religious beliefs that encourage having more than one wife are perceived to promote MCSP (57, 70).

Beyond the social and cultural factors, there are institutional or health system factors that facilitate MCSP among adolescents and should not be overlooked. Studies indicate that health worker attitudes and inadequate communication skills for reaching adolescents discourage adolescents from seeking information and services related to sexuality and MCSP (40, 51). Coupled with this is the inadequate availability of medical supplies in health facilities and inadequate quality of adolescent SRH services provided in Africa (51). Structural barriers also aggravate the challenge of adolescent access to SRH services. Distances to health facilities, poor quality of health services, and the cost of SRH services discourage adolescents from seeking health care services and information related to MCSP (30, 35, 51). The unfavorable policies on adolescent access to SRH services and limited gender equity laws constrain adolescents' access to services and increase gender power imbalances (30, 51). Figure 1 summarizes the main findings identified in the empirical literature on MCSP among adolescents.

### Theoretical Support for the Determinants of Adolescent MCSP (Step 4 of Core Processes)

Supportive theoretical constructs were reviewed to generate possible answers to what is known about the problem of MCSP among adolescents. Using the topic approach, theoretical constructs from empirical studies were identified and reviewed in the current study. Six theories identified from other studies included: Theories of Reasoned Action and Planned Behavior (71–74), Health Belief Model (75, 76), Social Cognitive Theory (15, 77, 78), Social Exchange Theory (14), Social Ecological Model (30, 32, 33), and Social Norms Theory (79–82). In the concept formation approach, the ideas generated from the brainstorming session and empirical literature were grouped and relabeled using these six theories. Labels such as attitude, perceived susceptibility beliefs, self-efficacy, and social/subjective norms were drawn from the aforementioned theories.

Using the general theories approach, we reviewed theories that offered detailed explanations and provided potential answers to the problem of MCSP among adolescents to gain additional insights for explaining the problem. We explored alternative theoretical frameworks that were not identified at the topic and concept approach steps but deemed relevant for the MCSP problem. Two alternative theoretical frameworks explored included the Self-Regulation Theory and the Extended Parallel Process Model. To better understand and group similar theoretical constructs in this study, all eight theories identified for review were classified into three categories: (1) individual, (2) community, and (3) environmental theories. Individual-level theories included Theories of Reasoned Action and Planned Behavior, Health Belief Model, Social Cognitive Theory, Extended Parallel Process Model, and the Self-Regulation Theory. Community-level theories included The Social Norms Theory and the Social Exchange Theory. Environmental-level theories only included The Social Ecological Model. To avoid replication, our review did not include theories that advanced constructs already known and identified in the brainstorming sessions and empirical literature. Only theories that highlighted new and additional insight into the problem of MCSP (four in total) were reviewed and documented for application to this study (Table 2).

**Table 2:** Theoretical support at the individual and community levels and its application to the problem of MCSP among adolescents in Uganda.

**Theoretical support**	**Main theoretical constructs**	**Application to MCSP among adolescents**
**INDIVIDUAL THEORIES**		
Extended Parallel Process Model (83–88)	The model advances constructs of fear/threat, efficacy, and responses to danger in relation to susceptibility and self-efficacy. Predicts that fear-arousing persuasive messages should show an effect on behavior only if both efficacy and threat are successfully manipulated.	Proposes the use of risk messaging to elicit fear or danger control responses for those who perceive a low risk and susceptibility to HIV. It is imperative to note that sometimes the use of risk messaging to elicit fear may not work if both components of fear and efficacy are not considered. Therefore, the model helps explain the perceived low risk and susceptibility to HIV among adolescents.
Self-Regulation Theory (89–91)	Self-regulation refers to self-generated thoughts, feelings, and actions that are planned and cyclically adapted to the attainment of personal goals.	Delineates aspects of self-motivation such as self-efficacy, goal setting, self-judgment, and self-evaluation. Adolescents engaging in MCSP were found to have low self-efficacy and there is limited information on their motivation to engage in MCSP.
**COMMUNITY THEORIES**		
Social Norms Theory (79–81)	States that our behavior is influenced by incorrect perceptions of how other members of our social groups think and act (misconception).	Outlines the pertinence of presenting communally accepted norms that exist in support of existing policies. This is an indication that communally accepted norms, such as gender equity and discouraging early marriages—both of which discourage MCSP—need to be grounded in policy.
Social Exchange Theory (14, 92, 93)	Delineates a two-sided notion of mutually contingent and rewarding processes involving “transactions” or simple “exchange.”	Indicates that the recipient of a gift is somehow obligated to provide a return. Therefore, adolescents' rational sense of action to engage in MCSP may be based upon a prior calculation of expected returns.

*MCSP, Multiple Concurrent Sexual Partnerships*.

#### Individual-Level Theories

Theoretical support on an individual level for the Extended Parallel Process Model indicates that peoples' perceptions of a threat often draw them to action. The model proposes the use of risk messaging to elicit fear and stimulate danger control responses (83–87, 94). However, to be effective, risk messages must contain both a threat component that creates a perception of personal susceptibility and severity and an efficacy component that provides information about measures to employ in reducing the threat (84, 85, 88). Many interventions that attempted to use a risk-messaging approach were found to be unsuccessful because they tended to focus on the fear component and leave out efficacy (88). Thus, they failed to meet the requirements as specified within the model. This model helps explain and contextualize the perception of low-risk and susceptibility to HIV among adolescents in Uganda.

The Self-Regulation Theory postulates that self-regulation refers to self-generated thoughts, feelings, and actions that are planned and cyclically adapted to the attainment of personal goals (89–91). The theory delineates aspects of self-motivation to perform a behavior such as self-efficacy, goal setting, self-judgment, and self-evaluation. These self-motivated actions may be critical in further understanding sexually active adolescents' low level of self-efficacy and motivation for having MCSP. It is important to note that motivation might not be driven only by the self, but also driven by other factors within one's environment that may influence a decision to engage in the behavior.

#### Community-Level Theories

The community-level Social Norms Theory describes the situation in which individuals incorrectly perceive the attitudes and/or behaviors of peers and community members to be different from or similar to their own (79–81). The theory proposes that presenting communally accepted norms—such as promoting gender equity and discouraging early marriages—that exist in support of existing policies are pertinent. This implies that to understand the problem of MCSP among adolescents, we need to know the policy environment in which communally accepted norms for behavior change are found.

The Social Exchange Theory advances actions that are contingent on rewarding reactions from others (92). The theory mainly positions reciprocity or repayment in kind as the main exchange rule (93). The notion that the recipient of a gift is somehow obligated to provide a return gives more insight into understanding why adolescents engage in multiple transactional sexual relationships. Table 2 summarizes the theoretical support and application to the study.

### Identified and Addressed the New Needs for Research on Adolescent MCSP

#### Missing Links in the Empirical Literature on Adolescent MCSP (Step 5 of Core Processes)

The study identified the missing links in the empirical literature about adolescent MCSP to address new research needs. Despite existing empirical literature on adolescent MCSP, knowledge on why adolescents engage in MCSP is still limited. Such gaps continue to hinder the design of effective adolescent health-targeted programs addressing sexual behavior change. Missing links categorized by individual determinants and environmental agents were identified from the empirical literature reviewed and theoretical support of the four alternative theories (Table 2).

##### Individual Determinants

Even with the existing studies about sexual relationships of adolescents in Africa, there is still limited research on MCSP and interventions targeted toward reducing MCSP among adolescents (95, 96). Although studies found that males are more likely to have multiple concurrent sexual partners than females, it is still unclear why adolescents with HIV are more likely to be female than male if MCSP increases the risk of contracting HIV (1, 38). It is also not yet clear why adolescents attending family planning clinics are eager to address their reproductive concerns, but appear to be less concerned about STDs (36, 37). This indicates an urgent need to discover why adolescents continue to risk contracting HIV through sexual activity.

We did not find literature that addressed new research at the community level, but new research needs were identified with environmental agents.

##### Environmental Agents

In Uganda, little is known about the motivations behind sexual behavior among adolescents and how these might differ by background factors such as gender and age. It is thus important to explore whether adolescents with an earlier sexual debut have unique attitudes or beliefs that make them more likely to have many sexual partners than those who delay sexual debut (1, 97, 98). Gender differences in multiple concurrent sexual relationships need to be clearly assessed for targeted programming (39). It is not clear in the literature whether relationships, wherein a woman is younger than her sexual partner, are always characterized by sexual coercion or not, yet older men and women often engage with adolescent girls and boys with the belief that they are “safe” from infection (40, 41, 97). Many studies on MCSP report the limitation of under-reporting and under-measurement of multiple sexual partners among adolescents (3, 35, 39). This indicates the need to re-examine the role of MCSP and the resulting sexual networks in HIV transmission (8). The ultimate design of interventions should be able to impact on adolescents' reference or peer group norms, which have been found to influence sexual behaviors such as MCSP (38). Emotional aspects of relationships and ethnic cultural differences are often overlooked in research and practice on adolescent health in Africa. It is important to explore how other factors such as love, trust, and commitment are associated with MCSP among adolescents (14, 38). Table 3 summarizes the missing links in the empirical literature about MCSP among adolescents by individual determinants and environmental agents.

**Table 3:** Missing links in the empirical literature and recommendations for further research on adolescent MCSP.

**Main issues for MCSP**	**Missing links and recommendations for research on adolescent MCSP**
**INDIVIDUAL DETERMINANTS**	
Attitude	• Need to explore whether adolescents with an earlier sexual debut have unique attitudes or beliefs that make them more likely to have many sexual partners, or have had a longer time period to accrue partners (34). • It is important to clarify the extent of HIV risk through multiple sex partners among adolescents by quantifying the sequence and frequency of sexual partners (34, 35).
Risk perception and beliefs about susceptibility to HIV	• Need to examine why adolescents attending family planning clinics are eager to address their reproductive concerns but appear to be less concerned about STDs (36, 37). • It is unclear why adolescents with HIV are more likely to be female than male (1, 38).
Self-efficacy	• Little is known about the motivations behind sexual behavior among adolescents and how these might differ by gender and age (34, 36, 39).
**ENVIRONMENTAL AGENTS**	
Gender and social norms	• Gender differences in concurrent relationships need to be clearly assessed for targeted programming (36, 39).
Communication with parents	• Little is known about how parents can communicate with and influence their adolescent children (40, 41). • Little is known about how couples communicate, interact and resolve problems, especially with regard to sexual health (14).
Other influencing factors	• It is important to further explore the influence of other factors such as trust, the social embarrassment when buying condoms, and sub-cultural differences with respect to sexual values (38). • Understanding how the quality of relationships, such as love, trust, and commitment, influence engagement in MCSP is pertinent (14).

*MCSP, Multiple Concurrent Sexual Partnerships; STDs, sexually transmitted diseases*.

#### Specific Research Questions

From the missing links in existing literature, we identified specific research questions for further exploration. These include:

- How do the patterns of sex partners among adolescents compare to those of the adult population?
- Why are adolescents more concerned about pregnancy than the risk of STDs and HIV?
- How do the attitudes and beliefs of adolescents with an early sexual debut influence their likelihood to have MCSP?
- What are the specific ethnic cultural, gender, and social norms that precipitate MCSP among adolescents in Uganda?
- How do MCSP among adolescents increase HIV transmission in the resultant sexual networks?

#### Proposed Answers for Reducing the Problem of Adolescent MCSP (Step 6 of Core Processes)

The main findings from the brainstorming sessions, empirical literature, and theoretical support indicate that it is important for health promotion programs to address the recurrent factors that influence adolescents to engage in MCSP. These include attitude, perceived susceptibility, perceived social/subjective norms, self-efficacy, knowledge, communication with partners, and other influencers, and social exchange/transactional sexual relations. The recurrent individual and environmental determinants identified in the brainstorming sessions, empirical literature and supported by theory were ranked by importance and changeability. Importance refers to the determinants that contribute significantly to the behavior, and changeability refers to the determinants that can be changed with the available methods or interventions (15, 25). The ranking helped in identifying highly important and changeable determinants to guide future design of adolescent health interventions using the currently available evidence. Determinants that were found to be important and changeable were ranked using ++ = very important and ++ = it is changeable. Table 4 summarizes the rankings of the identified list of determinants that provide possible answers/explanations to the problem of MCSP among adolescents. It is important to note that we did not find community-level answers to the problem.

**Table 4:** List of determinants providing possible answers for reducing adolescent MCSP.

**Determinants**	**Importance**	**Changeability**
**INDIVIDUAL DETERMINANTS**		
Attitude—Individual	++	++
Perceived susceptibility and risk to HIV	++	++
Self-efficacy	++	++
Perceived social/subjective norm	+	++
Knowledge of the risks of MCSP—Individual	+	++
Communication with sexual partner and influencers	++	++
Social exchange/transactional sexual relations	+	+
**ENVIRONMENTAL AGENTS**		
Attitude – Influencers	+	++
Knowledge of the risks of MCSP – Influencers	+	++
Social/group norms	+	+
Perceived social/subjective norm	+	++
Communication with parents	++	++
Communication with sexual partner	++	++
Social exchange/transactional sexual relations	+	+
Policy environment targeting adolescent access to SRH	++	+

*++, very important, and it is changeable; +, important, and may be changed; MCSP, Multiple Concurrent Sexual Partnerships; SRH, Sexual and Reproductive Health*.

## Discussion

This study explored why adolescents in Uganda engage in MCSP. The findings will guide the design of targeted adolescent SRH programs in Uganda. The findings from the ranked determinants delineate several factors associated with MCSP among adolescents at various levels of influence. The determinants ranked by importance and changeability generated a list of possible answers for designing targeted interventions that address the problem of adolescent MCSP. However, this list should be interpreted with caution, because it is not exhaustive. The list indicates what is known in the currently available literature. So, future interventions should rely on currently available evidence.

The findings indicate that adolescents engage in MCSP because they perceive low risk and susceptibility to HIV (34, 38). With this perception, adolescents fail to see the risk associated with their sexual behavior (67). Poor communication with sexual partners and parents was found to be associated with adolescent MCSP both in the brainstorming sessions as well as in the literature (41, 60–63). However, Dolcini et al. found that people who are comfortable talking about sex may simply be better at getting new sexual partners than those who are poor communicators (67), and this might also be true for adolescents. This accentuates the need to further understand how adolescent couples communicate, interact and resolve problems, especially with regard to sexual health and MCSP (14). Knowledge of the risks of MCSP among adolescents and their influencers was found to be inadequate and often intertwined with myths and misconceptions (65). Although several studies on adolescent sexual behaviors found that knowledge is weakly associated with the performance of the actual behaviors (36, 65, 67), information services remain important in clearing misconceptions and addressing health concerns. Brainstorming sessions and the literature showed that adolescents have low levels of self-efficacy toward having one sexual partner, often associated with the perception that peers have multiple sexual partners (34, 38). Community influences, including societal approval of MCSP and beliefs about the sexual dominance of men (55, 66, 70), were found to increase permissive attitudes of adolescents and their influencers on infidelity (50, 57, 67). Transactional sexual relationships whereby adolescents engage with older men and women called “sponsors” were found to be widespread (40, 51–54). Studies argue that the notion of transactional sexual relationships is deeply rooted in cultural contexts that model sexual relationships of exchange on traditional social institutions of courting and bride-wealth payments (55, 56). With these social influences, social norms on MCSP including gender equity and discouraging early marriages, communally deemed as appropriate, need to be grounded in policy, and effectively communicated to the general public (58, 65, 79, 81). The unfavorable policy environment surrounding adolescent access to SRH contributes to the problem of adolescents engaging in MCSP (30, 51). It is important to work with and form strategic partnerships with multiple key players in the institutional and structural environment to create a conducive environment for adolescent health.

Even with the existing empirical literature, there are still unanswered questions and missing links that need to be addressed to increase the understanding of why adolescents engage in MCSP. Not yet known are sexual relationship patterns among adolescents (95, 96), and why adolescents are more concerned about unplanned pregnancy than HIV risk (36, 37). Several studies in Uganda have reported that adolescents are increasingly becoming sexually active, depicted by early sexual debut since many adolescents report starting sex as early as 14 and 15 years (1, 2). However, it is not yet clear whether an early sexual debut eventually influences MCSP among adolescents (1, 34, 97, 98). The gap in understanding gender differences in multiple concurrent sexual relationships remains a challenge (36, 39). It is also not yet clear whether relationships in which an adolescent is younger than his or her sexual partner are characterized by violence and sexual coercion. It is imperative to understand the power balance dynamics in all sexual relationships, including MCSP in the light of married and unmarried adolescents, and those who are in-and-out of school (36, 39, 53, 58). There is inadequate documentation of the sequence and frequency of sexual partners, coupled with underreporting of sexual partners among adolescents (5, 35). Other factors including ethnic cultural differences with respect to sexual values, and emotional aspects including love, trust, and commitment have only been studied to a limited extent and data are needed to understand how they influence adolescent MCSP (14, 38). These gaps are a clear indication that additional research is needed to further understand the missing links on why adolescents engage in MCSP. Studies recommend longitudinal research to understand the complex notion of and ever-changing sexual relationships of adolescents particularly in sub-Saharan Africa (5, 9, 38).

Despite the gaps in the existing empirical literature, reviewed studies provided relevant recommendations for designing targeted adolescent health programs. Several studies highlighted the need to intensify sexuality education and strengthen communication skills for adolescents and their influencers (34, 38, 65, 67). Health-promotion programs need to communicate the importance of using condoms and how to use and enjoy sex with condoms to reduce the HIV risk among adolescents (56, 66). The use of mass media coupled with face-to-face interaction and public dialogue is pertinent for reinforcing social-change interventions (82). Gender integration should be central to health promotion to address inequitable socio-cultural norms that encourage adolescents to engage in MCSP (42, 49, 68, 69). It is vital to engage influential cultural institutions to promote longer courting for adolescents in relationships as a way of encouraging greater commitment to one relationship and to deter casual and concurrent sexual partnerships (55, 56). Theoretical support drawn from the Social Norms Theory indicates that to address social norms, it is important to develop norms grounded in policy (80, 81). Therefore, health promotion programs need to partner with governments who have a role in legitimizing normative change efforts and work within a policy context (82). Rather than solely depending on national surveys such as Demographic Health Surveys, program development practitioners need to devise other routine methods of monitoring the sequence and frequency of MCSP among adolescents in regard to HIV transmission (35).

## Study Limitations

This study generated insights from group brainstorming sessions and empirical literature review of MCSP studies from countries other than Uganda. This may limit the generalizability of the findings to Uganda's population. However, additional studies of a similar nature can be conducted in Uganda to offer a contextual comparison of the findings. The few sub-cultural contexts described in this study might not provide a deeper understanding of the multi-cultural divides in Uganda. Therefore, it is imperative to conduct further studies to understand the problem of adolescent MCSP within the confines of the multiple ethnic cultural settings in Uganda. Although the study used core processes including theoretical support to generate a list of possible answers for why adolescents engage in MCSP, this list is not exhaustive or conclusive in addressing the problem. This means that there might be other contributing factors that the currently available literature may not have captured.

## Conclusion

MCSP among adolescents in Uganda is a problem attracting the attention of international and local development partners who are eager to understand why there is increasing HIV/AIDS among this population. Our findings indicate that programs targeted to reduce the number of sexual partners among adolescents in Uganda should strive to design integrated interventions that address the determinants of MCSP at various levels of influence. The findings not only show the need to work with adolescents to understand their contextual and emerging health communication needs to appropriately position the risk of MCSP messaging, but they also indicate the need to involve and partner with other key players. Therefore, forming strategic partnerships with adolescents' families, communities, cultural, and religious institutions, health facilities, and local governments for concerted efforts to address the problem of MCSP is critical.

## Author Contributions

JN led the conceptualization and technical writing of the manuscript, led in empirical literature extraction and review, and the study design; JA, BB, PB, and RC contributed to the conceptualization of this study, empirical literature extraction, and review, and guided the structuring and technical writing of the manuscript. All authors reviewed and approved the final version of the manuscript.

### Conflict of Interest Statement

The authors declare that the research was conducted in the absence of any commercial or financial relationships that could be construed as a potential conflict of interest.

## Abbreviations

MCSP: Multiple Concurrent Sexual Partnerships
SRH: Sexual and Reproductive Health
STDs: Sexually Transmitted Diseases
HIV: Human Immunodeficiency Virus.
